# Current News

**Published:** 1893-03

**Authors:** 


### Current News.

### ANNUAL MEETING OF THE INTERNATIONAL DENTAL PUBLISHING COMPANY.

The annual meeting of the stockholders of the International Dental Publishing Company took place at Taylor's Hotel, Jersey City, N. J., on the evening of January 28, 1893.

Representatives were present from New York, Boston, Chicago, Philadelphia, and neighboring places.

The report of the president, Dr. Louis Jack, gave a very full and satisfactory exhibit of the condition of the company, together with that of the International Dental Journal, during the past year, 1892, and made various suggestions of importance for the future advancement of the work.

The resignation of Dr. Joseph Head as assistant editor was received, and accepted with regret, and a resolution of thanks was extended him for his services.

Dr. George W. Warren, of Philadelphia, was elected to fill the position made vacant by this resignation.

The Board of Managers were authorized to permanently increase the size of the International Dental Journal to eighty pages.

They were also empowered to create a department for translations, a special sum being appropriated for this purpose.

The general feeling with those present was that the Journal had become indispensable as the organ of the dental profession and that its future must necessarily be dependent upon the interest taken in it by the dentists of the entire country. Its success thus far was felt to be indicative of a growing feeling that a journal independent of cliques, and representing the best standard, must be sustained. To accomplish this purpose it should enter every office in this and other lands; but this desirable end can only be gained by every one devoted to the interests of the dental profession working earnestly to enlarge its influence by increasing its circulation.

### WORLD'S COLUMBIAN DENTAL CONGRESS.

### NEW ORDER OF BUSINESS.

*August* 14, *Monday.*—10 A.M., Meeting of the General Executive Committee. 11 A.M., Opening of the Congress; reading of the resolutions creating the Congress by the Secretary-General. Address of welcome by John Temple Graves, of Georgia; responses; responses from foreign countries. Address of the President. Adjournment. 2.30 P.M., Papers to be read in the sections. 5 P.M., Adjournment.

*August* 15, *Tuesday.*—9 A.M., Clinics. 10 A.M., Meeting of the General Executive Committee. 12 M., Address before the whole Congress. 1 P.M., Adjournment. 2.30 P.M., Papers to be read in Sections. 5 P.M., Adjournment. 8 P.M., Bacteriological exhibit.

*August* 16, *Wednesday.*—9 A.M., Clinics. 10 AM., Meeting of the General Executive Committee. 12 M., Address before the whole Congress. 1 P.M., Adjournment. 2.30 P.M., Garden party. 8 P.M., Biology; lantern exhibit. 8 P.M., Conversazione.

*August* 17, *Thursday.*—9 A.M., Clinics. 10 A.M., Meeting of the General Executive Committee. 12 M., General address before the whole Congress. 1 P.M., Adjournment. 2.30 P.M., Papers before the Sections. 8 P.M., Public address under direction of World's Congress Auxiliary.

*August* 18, *Friday.*—9 A.M., Clinics at hospitals and at the Art Institute. 10 A.M., Meeting of the General Executive Committee. 12 M., General address before the whole Congress. 1 P.M., Adjournment. 2.30 P.M., Papers before the Sections. 8 P.M., Dinner to the whole Congress. (Subscriptions by members from the United States only.)

*August* 19, *Saturday.*—10 A.M., Visit in a body to the medical and dental exhibits at the World's Fair Grounds. 12 M., Closing addresses to the Congress. Luncheon by members in the restaurant. (Name to be supplied.)

---

### ANNOUNCEMENT OF THE FIRST PAN-AMERICAN MEDICAL CONGRESS.

SECTION announcement of the First Pan-American Medical Congress, to be held at Washington, D. C., September 5, 6, 7, and 8, 1893:

President, William Pepper, M.D., LL.D., 1811 Spruce Street, Philadelphia, Pennsylvania; Secretary-General, Charles A. L.

Reed, M.D., 311 Elm Street, Cincinnati, Ohio; Treasurer, A. M. Owen, M.D., 507 Upper Front Street, Evansville, Indiana; Chairman of Executive Committee, Dr. Henry D. Holton, Brattleborough, Vermont.

*Committee of Arrangements.*—Chairman, Samuel S. Adams, M.D., 1632 K Street, Washington, D. C.; Secretary, J. R. Wellington, M.D., 1416 Fifteenth Street, Washington, D. C. (Office, Arlington Hotel, Washington, D. C.)

SECTION ON ORAL AND DENTAL SURGERY.

*Honorary Presidents.*—Dr. José Joaquin Aguirre, Santiago, Chili; Dr. R. R. Andrews, Boston; Dr. E. A. Baldwin, Chicago; Dr. George Beers, Montreal, Canada; Dr. S. B. Brown, Fort Wayne; Dr. Emegdio Carillo, City of Mexico, Mexico; Dr. Wm. Carr, New York; Dr. H. B. Catching, Atlanta; Dr. Geo. J. Fredericks, New Orleans; Dr. Ricardo Gordon, Matanzas, Cuba; Dr. J. H. Hatch; San Francisco; Dr. A. O. Hunt, Iowa City; Dr. Louis Jack, Philadelphia; Dr. H. J. McKellops, St. Louis; Dr. Francis Peabody, Louisville; Dr. J. C. Storey, Dallas, Texas; Dr. J. Taft, Cincinnati; Dr. J. B. Willmot, Toronto, Canada.

*Executive President.*—M. H. Fletcher, M.D., D.D.S., 65 West Seventh Street, Cincinnati, Ohio.

*Secretaries.*—Dr. John S. Marshall (English-speaking), Chicago, Illinois; Dr. Ramón Campuzano (Spanish-speaking), Philadelphia, Pennsylvania; Dr. N. Etchepareborda (Tacuari 355), Buenos Ayres, Argentine Republic; Dr. Wilson, La Paz, Bolivia; Dr. Benicio de Sá, Rio de Janeiro, United States of Brazil; Dr. Luke Teskey, Toronto, Canada; Dr. Guillermo Vargas Parédes (Carrera 7, núm. 638), Bogota, Republic of Colombia; Dr. J. Luis Estrada, Guatemala City, Guatemala; Dr. George Herbert, Wailuku Maui, Hawaii; Dr. Rafael Rico (Escuela de Med.), City of Mexico, Mexico; Dr. A. Lacayo, Granada, Nicaragua; Dr. Andres G. Weber (Corrales 1), Havana, Cuba; Dr. Angel Guerra, Montevideo, Uruguay.

EXTRACTS FROM REGULATIONS AND BY-LAWS.

*Membership.*—Members of the Congress shall consist of such members of the medical profession of the Western Hemisphere, including the West Indies and Hawaii, as shall comply with the special regulations regarding registration, or who shall render service to the Congress in the capacity of foreign officers. [*General Regulation* 2.]

*Constituent Countries.*—The following shall be considered as the constituent countries of the Pan-American Medical Congress:

Argentine Republic, Bolivia, Brazil, British North America, British West Indies (including B. Honduras), Chili, Dominican Republic, Honduras (Sp.), Mexico, Nicaragua, Paraguay, Peru, Salvador, Republic of Colombia, Republic of Costa Rica, Ecuador, Guatemala, Hayti, Kingdom of Hawaii, Spanish West Indies, United States, Uruguay, Venezuela, Danish, Dutch, and French West Indies. [*General Regulation* 7.]

*Sections.*—The Sections of the Congress shall be as follows:

(1) General Medicine, (2) General Surgery, (3) Military Medicine and Surgery, (4) Obstetrics, (5) Gynæcology and Abdominal Surgery, (6) Therapeutics, (7) Anatomy, (8) Physiology, (9) Diseases of Children, (10) Pathology, (11) Ophthalmology, (12) Laryngology and Rhinology, (13) Otology, (14) Dermatology and Syphilography, (15) General Hygiene and Demography, (16) Marine Hygiene and Quarantine, (17) Orthopædic Surgery, (18) Diseases of the Mind and Nervous System, (19) Oral and Dental Surgery, (20) Medical Pedagogics, (21) Medical Jurisprudence, (22) Railway Surgery. [*General Regulation* 8.]

*Languages.*—The languages of the Congress shall be Spanish, French, Portuguese, and English. [*General Regulation* 9.]

*Registration.*—The registration fee shall be ten dollars for each member residing in the United States, but no fee shall be charged to foreign members. Each registered member shall receive a card of membership and be furnished a set of the Transactions. [*Special Regulation* 2.]

*Abstracts, Papers, and Discussions.*—Contributors are required to forward abstracts of their papers, not to exceed six hundred words each, to be in the hands of the secretary-general not later than the 10th of July, 1893. These abstracts shall be translated into English, French, Spanish, and Portuguese, and shall be published in advance of the meeting for the convenience of the Congress, and no paper shall be placed upon the programme which has not been thus presented by abstract. Abstracts will be translated by the Literary Bureau of the Congress at the request of contributors, and should be forwarded through secretaries of Sections. Papers to be presented to Sections must not consume more than twenty minutes each in reading, and when of greater length must be read by abstract not exceeding twenty minutes in length. Papers read by abstract may be printed in full in the Transactions, subject to approval by the Editorial Committee. Papers and discussions will be printed in the language in which they may be presented. All papers read in the Sections shall be surrendered to the secretaries of the Sections; all addresses read in the General Session shall be surrendered to the secretary-general as soon as read; and all discussions shall be at once reduced to writing by the participants. [*Special Regulation* 3.]

*Literary Bureau.*—The secretary-general may at his discretion organize a Literary Bureau, which shall consist of such number of linguists as he may determine, whose duty it shall be to do all necessary translating for the Congress, compensation for which service shall be determined by the Executive Committee. Certain members of the Literary Bureau may be designated by the secretary-general as an Editorial Committee. It shall be the duty of the Editorial Committee to determine the eligibility of all contributions before the same shall be published in the Transactions, and to supervise the publication of both the Book of Abstracts and the Transactions. All work done by the Editorial Committee and by the Literary Bureau shall be subject to approval by the secretary-general. [*By-law V.*]

---

### THE PAN-AMERICAN MEDICAL CONGRESS.

### SECTION ON DISEASES OF THE MIND AND NERVOUS SYSTEM—PRELIMINARY MANIFESTO OF THE SECTION OF NERVOUS AND MENTAL DISEASES OF THE PAN-AMERICAN MEDICAL CONGRESS OF 1893.

St. Louis, January 17, 1893.

To the Editor of the International Dental Journal:

Sir,—The Pan-American Medical Congress will meet in Washington September 5, 6, 7, and 8, 1893.

I take pleasure in transmitting herewith a manifesto of the preliminary organization of the important Section of Psychiatry and Neurology of the forthcoming Pan-American Medical Congress,[1] with request that you publish the same in your estimable journal, with editorial endorsement and cordial invitation to the medical profession of your section to co-operate in promoting the success of this section of the coming Congress, by suggestion, by offering papers to be read, by promptly signing as members, by

---

[1] The list of officers accompanying this is too extended for our space, but it covers many of the most prominent names in medicine on this continent.—Ed.

letters, and by advice to the executive president of the Section, or to its English-speaking secretary, Dr. A. B. Richardson, Columbus, Ohio.

Valuable papers have been promised from distinguished savants in neurological and psychological medicine, but many more are desired and desirable. The Spanish, French, and English languages will be spoken in the Section, and it is especially desired to secure as good a representation of the profession and make as good an exhibit of the advance in neurology and psychiatry as may be possible.

This, together with a desire for closer confraternity between the profession of the North and South American States, as well as the welfare of our common humanity, of which the approaching Congress will be promotive, are chief among the exalted purposes of this Section.

Physicians who may desire to identify themselves with this Section of the Congress are requested to do so at once.

Fraternally,

C. H. Hughes,

*Executive President Section on Diseases of the Mind and Nervous System, Pan-American Medical Congress.*

---

### RECENT PATENTS.

A list of recent patents, reported specially for The International Dental Journal:

488,634. Dental Filling. William D. Porter, Providence, R. I. Filed May 20, 1892.

489,117. Dental Engine. Frederick H. Berry, Milwaukee, Wis. Filed July 25, 1892.

489,235. Dental Apparatus. Alvan S. Richmond, New York, N. Y., assignor to John S. Huyler, same place. Filed June 12, 1891.

489,416. Dental Engine. Frank K. Hesse, Boston, Mass., assignor to Codman & Shurtleff, same place. Filed June 9, 1892.

489,675. Dental Chair. Aaron P. Gould, Canton, Ohio. Filed February 24, 1890.

490,516. Dental Chair. Eli T. Starr, Philadelphia, Pa., assignor to the S. S. White Dental Manufacturing Company, same place. Filed February 14, 1890.

Trade-Marks. 22,259. Dentifrices. Herman B. Fould, New York, N. Y. Filed November 19, 1892. Essential feature, the word "Ricealine."

22,260. Filling or Cement for Teeth. Robert Richter, Philadelphia, Pa. Filed November 28, 1892. Essential feature, the representation of a shield having the words "Harvard Cement" upon its face, with the abbreviation, word, and letters "Rob. Richter, D.D.S," also applied thereon.

22,296. Tooth- and Gum-Washes. James J. Ottinger, Philadelphia, Pa. Filed November 25, 1892. Essential feature, the word "Zhongiva."

---

### AMERICAN ELECTRO-THERAPEUTIC ASSOCIATION.

The third annual meeting of this organization will be held in Chicago on September 12, 13, and 14, 1893. A cordial invitation is extended to all members of the profession interested in electro-therapeutics. Arrangements for special rates on railways and at hotels are in progress.

The Committee of Arrangements will be obliged if those who intend being present at the meeting will send their names, the class and amount of accommodation required, titles of papers to be presented, applications for membership, etc., at as early a date as possible. Accommodation should be secured early on account of the crowded condition of the hotels, because of the World's Fair. All communications should be addressed to the Secretary.

The Committee will be glad to furnish any information in regard to the meeting upon application.

The Committee of Arrangements,
Franklin H. Martin,
*Chairman.*
S. C. Stanton,
*Secretary.*

---

### THE DOUCHE TOOTH-BRUSH.

The inventive genius in this direction was supposed to have been exhausted in the various forms presented in the market, but the above English device has opened further possibilities in this direction.

It consists of a small reservoir to be placed on the wall, from which leads a flexible rubber tube to be attached to the handle of the brush. This handle is perforated, and connected with small holes that open in the bristles. The object is to keep a constant irrigation of the brush when in use.

Theoretically it is a most excellent arrangement, and does its work thoroughly; but we anticipate difficulty will be found in controlling the stream of water, to prevent it running down the handle. In other respects it is a cleanly and efficient device. It is for sale by George B. Evans, 1106 Chestnut Street, Philadelphia.

---

### ALUMNI NEW YORK COLLEGE OF DENTISTRY.

At the annual meeting of the Alumni Association of the New York College of Dentistry, the following gentlemen were elected to office:

President, Sherman B. Price, D.D.S., 1880, 13 West Thirty-second Street, city of New York; First Vice-President, M. Charles Gottschaldt, M.D., D.D.S., 1882; Second Vice-President, H. J. Parker, D.D.S., 1883; Secretary, Vincent M. Munier, D.D.S., 1888, 102 West Ninety-fifth Street, city of New York; Treasurer, Zachary T. Sailer, D.D.S., 1880, 40 West Thirty-third Street, city of New York.

*Executive Committee.*—Chairman, J. Howard Reed, D.D.S., M.D.S., 120 West Eighty-seventh Street, city of New York; E. S. Robinson, D.D.S.; F. A. L. Wallin, D.D.S.

Vincent M. Munier,
*Secretary.*

---

### ODONTOGRAPHIC SOCIETY OF CHICAGO.

At the annual meeting of the Odontographic Society of Chicago, held at the Commercial Hotel, Monday evening, December 12, the following officers were elected for the ensuing year, viz.:

President, R. B. Tuller; Vice-President, G. J. Dennis; Corresponding Secretary, T. A. Broadbent; Recording Secretary, U. G. Poyer; Treasurer, Edmund Noyes.

*Board of Directors.*—R. B. Tuller, U. G. Poyer, H. H. Wilson, O. E. Meerhoff, D. M. Gallie.

*Board of Censors.*—G. N. West, F. K. Ream, F. H. Zinn.

T. A. Broadbent,
*Corresponding Secretary.*

### NORTHERN OHIO DENTAL ASSOCIATION.

The Thirty-fourth Annual Meeting of the Northern Ohio Dental Association will be held in the Council Chamber at Akron, Ohio, May 9, 10, and 11, 1893. Session begins at 10 A.M. Tuesday. You are cordially invited to be present.

---

### THE ST. LOUIS DENTAL SOCIETY.

The annual meeting of the St. Louis Dental Society was held at the office of Dr. J. B. Vernon, January 3, 1893. The following officers were elected for the ensuing year:

President, Dr. De Courcy Lindsley; Vice-President, Dr. J. Warren Wick; Corresponding Secretary, Dr. William Conrad; Recording Secretary, Dr. J. G. Pfaff; Treasurer, Dr. Henry Fisher.

*Committee on Publication.*—Dr. L. A. Young, Dr. P. H. Isloeffel, Dr. C. L. Pepperling.

*Committee on Ethics.*—Dr. H. M. Baird, Dr. A. J. Prosser, Dr. M. C. McNamara.

*Committee on Membership.*—Dr. W. N. Morrison, Dr. C. L. Hickman, Dr. J. H. Spalding.

Drs. A. H. Fuller, J. W. Wick, and W. M. Bartlett have been appointed a committee to arrange for a series of clinics in operative and mechanical dentistry, to be given in St. Louis on Wednesday, Thursday, and Friday, March 15, 16, and 17 next.

It is the intention of the Society to make this one of the most interesting and instructive meetings ever held in the West.

They want every reputable dentist in the country who can, to be present. There will be a chance to show what they have that is new, and see what others can do.

The clinics will be first-class from start to finish.

---

### SPECIAL NOTICE.

The St. Louis Dental Society will hold a three days' clinic March 15, 16, and 17, 1893. A general invitation is given for all dentists to attend. The committee having charge of the clinic already promise an interesting meeting. Drs. A. H. Fuller, J. Warren Wick, and W. M. Bartlett compose the committee.

Wm. Conrad,
*Corresponding Secretary.*

### OHIO STATE DENTAL SOCIETY.

At the Ohio State Dental Society, held at Columbus, December 6–9, 1892, the officers elected were as follows:

President, G. H. Wilson, Cleveland; First Vice-President, Chas. Welch, Wilmington; Second Vice-President, W. H. Todd, Columbus; Secretary, L. P. Bethel, Kentucky; Assistant Secretary, Henry Barnes, Cleveland; Treasurer, Chas. J. Keely, Hamilton.

L. P. Bethel,
*Secretary.*

